# Discovery of Novel Host Molecular Factors Underlying HBV/HCV Infection

**DOI:** 10.3389/fcell.2021.690882

**Published:** 2021-08-12

**Authors:** Xubo Huang, Joseph T. Glessner, Jinxia Huang, Desheng Zhou, Michael E. March, Hongna Wang, Qianghua Xia, Hakon Hakonarson, Jin Li

**Affiliations:** ^1^Affiliated Cancer Hospital and Institute of Guangzhou Medical University, Guangzhou, China; ^2^Key Laboratory for Cell Homeostasis and Cancer Research of Guangdong Higher Education Institutes, Guangzhou Medical University, Guangzhou, China; ^3^Center for Applied Genomics, The Children’s Hospital of Philadelphia, Philadelphia, PA, United States; ^4^Department of Pediatrics, The Perelman School of Medicine, University of Pennsylvania, Philadelphia, PA, United States; ^5^Division of Human Genetics and Division of Pulmonary Medicine, The Children’s Hospital of Philadelphia, Philadelphia, PA, United States

**Keywords:** hepatitis infection, autophagy, immune response to virus, genome-wide association study, microarray

## Abstract

Hepatitis is an inflammatory condition of the liver, which is frequently caused by the infection of hepatitis B virus (HBV) or hepatitis C virus (HCV). Hepatitis can lead to the development of chronic complications including cancer, making it a major public health burden. Co-infection of HBV and HCV can result in faster disease progression. Therefore, it is important to identify shared genetic susceptibility loci for HBV and HCV infection to further understand the underlying mechanism. Through a meta-analysis based on genome-wide association summary statistics of HBV and HCV infection, we found one novel locus in the Asian population and two novel loci in the European population. By functional annotation based on multi-omics data, we identified the likely target genes at each novel locus, such as *HMGB1* and *ATF3*, which play a critical role in autophagy and immune response to virus. By re-analyzing a microarray dataset from Hmgb1^–/–^ mice and RNA-seq data from mouse liver tissue overexpressing *ATF3*, we found that differential expression of autophagy and immune and metabolic gene pathways underlie these conditions. Our study reveals novel common susceptibility loci to HBV and HCV infection, supporting their role in linking autophagy signaling and immune response.

## Introduction

Hepatitis is an inflammatory condition of the liver, which can be an acute or a chronic infection. The most common types of hepatitis are caused by hepatitis B virus (HBV) or hepatitis C virus (HCV), both of which are major types of infectious disease affecting millions of patients and can lead to the development chronic complications such as cirrhosis or liver cancer (Schweitzer et al., 2015; Manns et al., 2017; Spearman et al., 2019). Therefore, it is important to understand the genetic and molecular mechanism underlying hepatitis viral infection for disease prevention and control. The contribution of host genetic factors is an important perspective of susceptibility and persistence of hepatitis infection (Ganem and Prince, 2004; Rehermann and Bertoletti, 2015). Genetic studies, especially genome-wide association studies (GWAS), in the last decade have identified a number of genetic susceptibility loci for HBV (Zhang et al., 2019b) and HCV (Yan and Wang, 2017). Most of the GWASes for HBV were conducted in Asian populations and those for HCV were conducted in European populations.

Though HBV and HCV are due to infection by different types of viruses, and they have differences in transmission and symptoms, they share certain common underlying molecular mechanisms (D’souza et al., 2020). The two types of virus can be co-transmitted, and some patients have co-infection of HBV and HCV simultaneously, which leads to acute fulminant hepatitis and chronic hepatitis. The worldwide prevalence rate for HBV and HCV co-infection is estimated to be 1–15% (Mavilia and Wu, 2018).

It is important to understand the shared host genetic and molecular factors for HBV and HCV within different populations for the purpose of disease prevention and control. To address these questions, we carried out a comparative study based on the GWAS data reported in the Pan UK Biobank, Japanese Biobank, and those in Gene Expression Omnibus (GEO). We found genes functioning in the autophagy pathway are among target genes of the shared genetic loci of HBV and HCV infection. A re-analysis of microarray data in a GEO dataset showed that deletion of target gene *Hmgb1* in mouse hepatic tissue resulted in differential expression of genes associated with autophagosome–lysosome fusion and perturbation of immune response to viruses. Re-analyzing another RNA-seq dataset from mice hepatic tissue overexpressing *ATF3* gene similarly demonstrated the significant change in the expression level of autophagy and metabolic genes. Our results suggest the role of autophagy-related genes in the common pathogenesis mechanism of HBV and HCV infection.

## Materials and Methods

### GWAS Data of Hepatitis C and Hepatitis B Virus Infection

The GWAS summary statistics were downloaded from the website of the Japanese ENcyclopedia of GEnetic associations by Riken,^1^ including the datasets of chronic hepatitis B and chronic hepatitis C. The GWAS summary statistics of the Pan UK Biobank were downloaded from https://pan.ukbb.broadinstitute.org/, including two datasets: viral hepatitis B and viral hepatitis C (Supplementary Table 1).

### Data Quality Control Filtering

Quality control (QC) filtering was performed on the summary statistics data of each cohort. Any variant with low confidence or minor allele frequency (MAF) < 0.01 was removed from further analysis. The number of variants before and after QC is shown in Supplementary Table 1.

### Data Analysis

After data QC, meta-analyses based on the above GWAS summary statistics of hepatitis infection were performed for each population. The *Z*-score-based method implemented in the software METAL (Willer et al., 2010) was adopted with sample overlap correction. This approach computed a weighted sum of *Z*-score for each effect allele of each single-nucleotide polymorphism (SNP) across cohorts, with weights defined by the square root of the effective sample size for each cohort. It also takes the direction of effect into account (Willer et al., 2010).

### Defining Novel Independent Association Loci

The associated SNPs were clumped into linkage disequilibrium (LD)-independent genomic regions using the software PLINK (Purcell et al., 2007) with the following parameter settings: –clump-*r*^2^ = 0.4, –clump-kb = 500, –clump-p1 = 5e-08, and –clump-*p*^2^ = 5e-02. LD information from the 1,000 Genomes phase 3 data (Auton et al., 2015) of European and East Asian ancestry was used in the analysis. The MHC region (chr6: 25–35 MB) was considered as one locus. The resulting independent genomic loci were compared to those reported in the GWAS catalog for hepatitis C and hepatitis B diseases. Any locus within 1 MB of a reported locus was regarded as a known locus. By excluding known loci, the remaining genome-wide significant (GWS) loci were considered as novel association loci.

### Fine Mapping

Fine mapping *via* the software FINEMAP (Benner et al., 2016) was conducted to identify variants in the 95% credible set of each novel GWS locus, with LD score calculation based on the EUR and EAS samples of the 1,000 Genome phase3 panel. The maximum number of allowed causal SNPs is set as 10 in the analysis. Causal variants were defined as those with *r*^2^ > 0.6 with the index SNP and the association *P*-value < 1 × 10^–4^ in the meta-analysis.

### Functional Annotation

Functional annotation of each GWS locus was performed with FUMA GWAS (Watanabe et al., 2017). Annotation of the expression quantitative trait loci (eQTLs) was based on scRNA eQTLs (van der Wijst et al., 2018), DICE (Schmiedel et al., 2018), eQTLGen, blood eQTLs (Westra et al., 2013), and GTEx v8 (Battle et al., 2017). The threshold for significant eQTL SNP–gene pairs was defined as a false discovery rate (FDR) < 0.05. To annotate chromatin interactions, the liver Hi-C datasets were analyzed by using the 3D Genome Browser and 3D-genome Interaction Viewer (Lieberman-Aiden et al., 2009; Wang et al., 2018; Yang et al., 2018).

### Microarray Data Analysis

The microarray dataset GSE49824 was downloaded from the GEO database (Huebener et al., 2014). The dataset contains gene expression profiling data from mouse hepatic stellate cells from four mice with hepatocyte-specific deletion of Hmgb1 and four control mice. The R package GEOquery was used for data download (Davis and Meltzer, 2007). Calibration, quantile normalization, and differential expression analysis were conducted with the R package limma (Ritchie et al., 2015). The significant genes were defined as log_2_| fold change (FC)| > 0.5 and *P*-value < 0.05.

### Differential Expression Analysis of RNA-Seq Data

Count data were downloaded from the GEO dataset GSE148301 (Xu et al., 2021). Then, the variance-stabilizing transformation (vst) normalization and differential expression were performed *via* the R package DESeq2 (Love et al., 2014). The significant genes were defined as log2| fold change (FC)| > 1 and *P*_adj_ < 0.05.

### Pathway Enrichment Analysis

The online web portal DAVID (Huang et al., 2007; Huang da et al., 2009b) was used for pathway enrichment analysis with differentially expressed genes as input. The collection of GO and KEGG pathway datasets was used to find significantly enriched pathways (Huang da et al., 2009a).

### Protein–Protein Interaction Network Analys**is**

The protein–protein interaction (PPI) network was predicted using the Search Tool for the Retrieval of Interacting Genes (STRING)^2^ online database (Franceschini et al., 2013), removing interactions with a combined score < 0.4. Then, the PPI network was drawn using Cytoscape (version 3.7.2). The significant interactions were identified using Degree (Smoot et al., 2011). Finally, DAVID was used to conduct pathway enrichment analysis on the 19 genes that were most closely related in the network (Huang da et al., 2009a).

### The Children’s Hospital of Philadelphia Replication Cohort

#### Study Subjects

Data for the replication cohort were extracted from the biobank of the Center for Applied Genomics (CAG), at the Children’s Hospital of Philadelphia (CHOP). Patients with a diagnosis of “Hepatitis C virus infection,” “Chronic hepatitis C,” “Hepatitis B virus infection,” or “Chronic hepatitis B” were selected for analyses. Age, sex, and ancestry-matched subjects without diagnosis of hepatitis were selected as controls. The ICD9 codes and the diagnosis name of the selected cases are listed in Supplementary Table 2.

#### Ethics Statement

The study was approved by the Institutional Review Board at CHOP, and written informed consent was obtained from all participating subjects and/or their parents. The study was carried out in accordance with the nationally approved guidelines.

#### Genotyping

Genomic DNA extracted from whole blood samples of the replication cohort was genotyped on Illumina HumanHap550-V1/V3, HumanHap610-Quad, or Infinium Global Screening Array-24 (GSA) DNA BeadChips at CAG.

#### QC Filtering

Samples with call rate < 98%, with ambiguous sex detected by PLINK (Purcell et al., 2007), or heterozygosity outliers were excluded. Duplicated or cryptically related samples were defined as PI_HAT ≥ 0.1875 in identity-by-state analysis and one from each pair was excluded. The SNPs to replicate were checked for genotype rate, minor allele frequency, and Hardy–Weinberg equilibrium test. All the QC steps were performed with PLINK.

#### Principal Component Analysis

Principal component analysis (PCA) was performed with PLINK to confirm the self-reported European ancestry and to derive the principal components, which were then applied to association analysis as covariates to control for population stratification.

#### Imputation

The unphased SNP-array genotype data of all the samples in the replication cohort were converted to VCF files and uploaded to Trans-Omics for Precision Medicine (TOPMed) imputation server^3^ for phasing using Eagle2 (Loh et al., 2016) and imputation using Minimac4^4^ (Fuchsberger et al., 2015) with the TOPMed r2 as the reference panel. After imputation, BCFtools v1.6 (Li et al., 2009) was used to remove SNPs with imputation score *R*^2^ < 0.3.

#### Association Analysis

Logistic regression was performed *via* PLINK to assess the association between SNP genotype and HBV/HCV status, including sex and the first 10 principal components as covariates.

## Results

### Identification of Three Novel HBV/HCV Susceptibility Loci From Meta-Analysis

**FIGURE 1:**
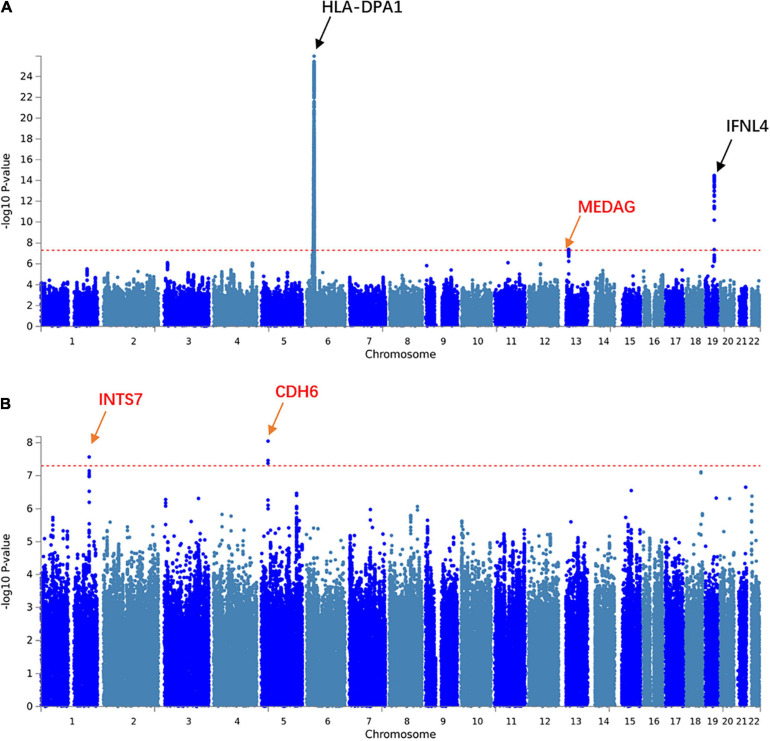
Manhattan plots showing the association statistics for hepatitis B virus (HBV)/hepatitis C virus (HCV) meta-analysis results in populations of Asian and European ancestries, respectively. **(A)** A meta-analysis on genome-wide association study (GWAS) summary statistics in the database of the Japanese ENcyclopedia of GEnetic associations by Riken. **(B)** A meta-analysis on GWAS summary statistics in the Pan UK Biobank. The genomic coordination of single-nucleotide polymorphisms (SNPs) is indicated on the *X*-axis, and the -log10 of *P*-value per SNP in meta-analysis is indicated on the *Y*-axis. The horizontal red line represents the genome-wide significance threshold *P*-value = 5 × 10^–8^. The closest genes to the previously reported loci are shown in black, and those closest to the novel loci are shown in red.

**FIGURE 2:**
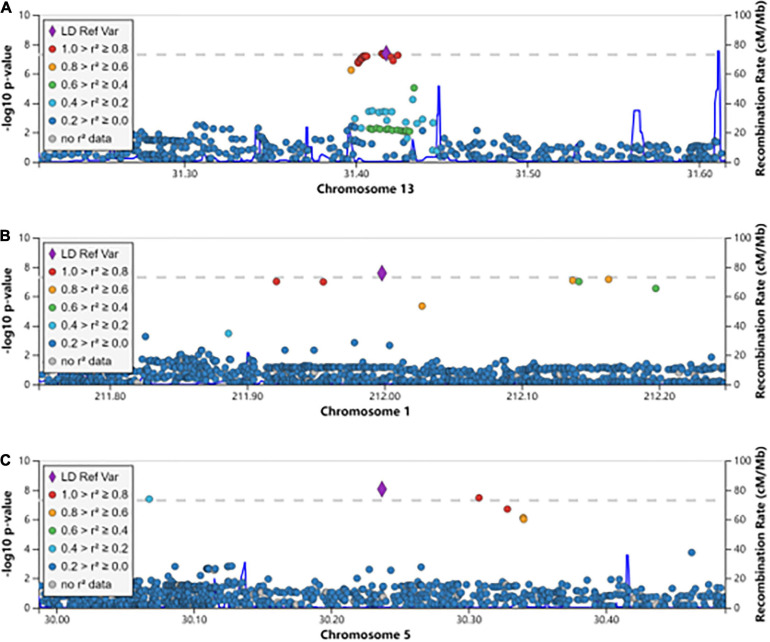
The regional association plots for the novel genome-wide significant loci associated with HBV/HCV infection. The genomic coordination is indicated on the *X*-axis, and the -log10 *P*-value of meta-analysis is shown on the left *Y*-axis. The most significantly associated SNP is shown in purple, and the different colors represent the linkage disequilibrium between the surrounding SNPs and the lead SNP. **(A)** The locus 13q12.3; **(B)** the locus 1q32.3; and **(C)** the locus 5p13.3.

We aimed to understand the common pathogenesis mechanism of HBV and HCV infection, to aid in protection from harmful coinfection of these viruses. We carried out a meta-analysis between the GWAS results of HBV and HCV within populations of European ancestry and Asian ancestry, respectively (Supplementary Table 1). In the Asian population, the HLA locus on chromosome 6 showed strong association and another previously reported locus on chromosome 19 at the 5’ of *IFNL4* also reached GWS (Figure 1A). In addition, we identified a novel GWS locus at 13q12.3, located in 5’ of gene *MEDAG* (Figures 1A, 2A). From the meta-analysis of the GWAS summary statistics of HBV and HCV in the Pan UK Biobank, we found two novel GWS loci at 1q32.3 and 5p13.3 (*P*-value < 5 × 10^–8^). These loci are in the intron of gene *INTS7* and 3’ of gene *CDH6*, respectively (Table 1 and Figures 1B, 2B,C). *INTS7* encodes integrator complex subunit 7, and CDH6 is a type II cadherin. Looking into the association of the top SNP at the novel GWS loci with HBV and HCV infections separately, we found that the top SNP at each novel locus was associated at nominal significance level (*P*-value < 0.05) in the original GWAS statistics and showed consistent direction of effects (Table 1). They reached GWS level in the meta-analysis due to the synergistic contribution from both disease cohorts instead of being driven by HBV or HCV specifically.

**TABLE 1:** The novel significant associations with HBV/HCV infection.

Population	SNP	Chr	Pos	Alt/Ref	Allele freq	*Z*-score	Direction	HBV_P/HCV_P	Meta-*P*-val	Closest gene
East Asian	rs34780238	13	31,416,073	C/T	0.4976	5.477	+ +	2.12E-03/1.64E-06	4.34E-08	*MEDAG*
European	rs142568263	1	211,998,341	T/C	0.0195	5.559	+ +	4.96E-06/9.82E-04	2.71E-08	*INTS7*
European	rs76561592	5	30,237,010	G/A	0.0140	5.750	+ +	8.76E-06/2.28E-04	8.93E-09	*CDH6*

*SNP, rsID of each SNP; Chr, chromosome; Pos, position (hg19); Alt, alternative allele; Ref, reference allele; Direction, the direction of effects; Allele freq, alternative allele frequency; HBV_P, P-value in HBV GWAS; HCV_P, P-value in HCV GWAS; Meta-P-Val, P-value in meta-analysis.*

### The *Cis*-Regulatory Role of Causal Variants Revealed by Fine Mapping and Functional Annotation

Next, we tried to understand how the novel loci in each population contribute to the development of HBV/HCV infection by performing functional annotation of SNPs at the novel loci and examining potential target genes. It is known that the causal variant at each locus may not be the lead SNP, but a SNP in LD. Therefore, we performed fine mapping to identify likely causal variants satisfying the criteria of LD, association in meta-analysis, and Bayesian statistics posterior probability (Table 2). The results show that lead SNPs rs142568263 at 1q32.3 and rs76561592 at 5p13.3 are in the credible set of potential causal variants. Additionally, four SNPs and two SNPs are in the credible set of these two loci, respectively, each with a posterior probability of 1. For locus 13q12.3, there are only three SNPs with a posterior probability > 0.2. Nine of these SNPs overlap with histone modification marks, such as H3K4me1, H3K4me3, H3K27ac, and H3K9ac in liver tissue, suggesting their potential role as *cis*-regulatory elements regulating target gene expression (Supplementary Table 3).

**TABLE 2:** The credible sets of potential causal variants at each novel genome-wide significant locus.

Population	SNP	Chr	Pos	Alt	Ref	Allele freq	*Z*-score	Direction	Meta_*P*	Prob
East Asian	rs34780238	13	31,416,073	C	T	0.4976	5.477	+ +	4.34E-08	0.308
East Asian	rs3922904	13	31,405,422	C	T	0.4926	5.415	+ +	6.12E-08	0.220
East Asian	rs9579658	13	31,405,059	G	A	0.4923	5.408	+ +	6.36E-08	0.212
European	rs75651594	1	212,163,342	G	A	0.0218	5.388	+ +	7.14E-08	1
European	rs142568263	1	211,998,341	T	C	0.0195	5.559	+ +	2.71E-08	1
European	rs148691694	1	211,921,465	C	T	0.0223	5.327	+ +	1.00E-07	1
European	rs150857619	1	212,141,784	A	C	0.0251	5.325	+ +	1.01E-07	1
European	rs146104002	1	212,341,169	T	C	0.0203	4.681	+ +	2.86E-06	1
European	rs76561592	5	30,237,010	G	A	0.0140	5.75	+ +	8.93E-09	1
European	rs77470613	5	30,340,210	A	T	0.0148	4.891	+ +	1.00E-06	1
European	rs77160283	5	29,978,363	A	G	0.0161	5.04	+ +	5.44E-07	1

*SNP, rsID of each SNP; Chr, chromosome; Pos, position (hg19); Alt, alternative allele; Ref, reference allele; Allele freq, alternative allele frequency; Direction, the direction of effects; Meta_P, P-value from meta-analysis; Prob, marginal posterior inclusion probabilities.*

### eQTL and Hi-C Analyses Suggesting Candidate Target Genes at Each Novel Locus

The SNPs at each GWAS locus could regulate the expression of distant genes through chromatin interaction, in addition to the regulation of its closest gene (Smemo et al., 2014; Claussnitzer et al., 2015; Xia et al., 2016). To identify target genes of each novel association locus, we examined eQTL genes in liver or immune cell types. Gene *LPGAT1* is an eQTL gene for SNP rs150857619 at locus 1q32.3 with FDR < 0.05 (Supplementary Table 4). *LPGAT1* encodes lysophosphatidylglycerol acyltransferase 1, which may be involved in triacylglycerol synthesis and secretion in the liver. Mouse Lpgat1 knockout resulted in phosphatidylglycerol remodeling defects and led to oxidative stress, mitochondrial DNA depletion, and mitochondrial dysfunction. The Lpgat1^–/–^ mice developed diet-induced obesity and hepatopathy phenotypes (Zhang et al., 2019a). These results indicate the role of LPGAT1 in mitophagy, which is an important type of selective autophagy to maintain cellular homeostasis upon virus invasion. The expression level of additional genes was found to have nominal significant association with the top associated SNP or causal variants at each novel GWS locus (*P*-value < 0.05) (Supplementary Table 4).

**FIGURE 3:**
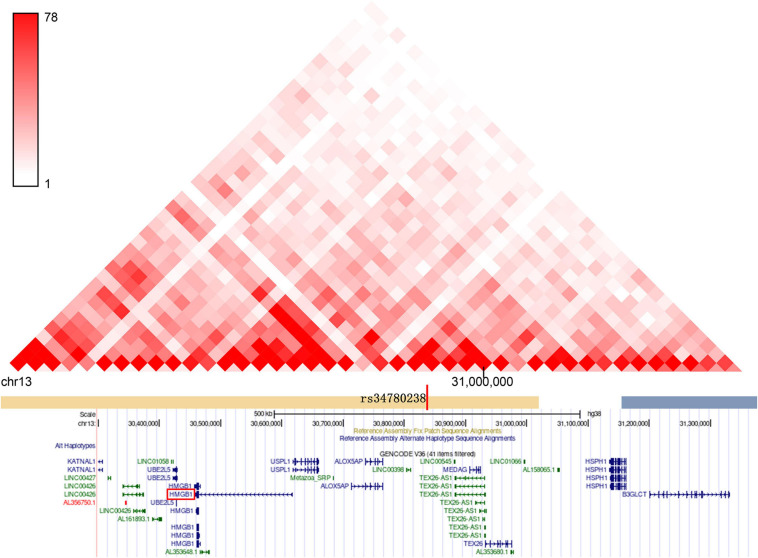
The genome-wide significant SNPs at the novel locus of 13q12.3 and its target genes are located in the same topologically associated domains (TAD).

We also examined genes having chromatin interactions with these loci in Hi-C datasets (Supplementary Table 5). The Hi-C data suggest that there is a chromatin interaction between the HBV/HCV susceptibility loci and the promoter region of multiple genes, which have been reported to be involved in autophagy and immune signaling pathways. For example, the target genes of locus 5p13.3: *CDH*6 inhibits autophagy and promotes re-organization of the mitochondrial network (Gugnoni et al., 2017); Drosha is involved in regulating hepatocyte growth (Fabris et al., 2019) and HBV replication control (Yang et al., 2017). *GOLPH3* is another targeted gene of locus 5p13.3 functioning in selective autophagy (Lu et al., 2020) and hepatitis C virus secretion (Bishe et al., 2012). Similarly, the target genes at locus 1q32.3: TRAF3IP3 enhances autophagy through interaction with an ATG16L1-binding motif (Peng et al., 2015), and ATF3 is a negative regulator of autophagy (Sood et al., 2017). Another gene of particular interest is *HMGB1*, which is in the same topologically associated domain (TAD) as SNP rs34780238 at 13q12.3 (Figure 3 and Supplementary Figure 1). Twenty three out of 54 Hi-C genes at these three loci exhibited nominally significant eQTL association with the three GWS loci, including the above autophagy-related genes *CDH6*, *ATF3*, and *HMGB1* (Supplementary Table 5).

### Alteration of Target Gene Expression Leads to Expression Change of Autophagy Genes

**FIGURE 4:**
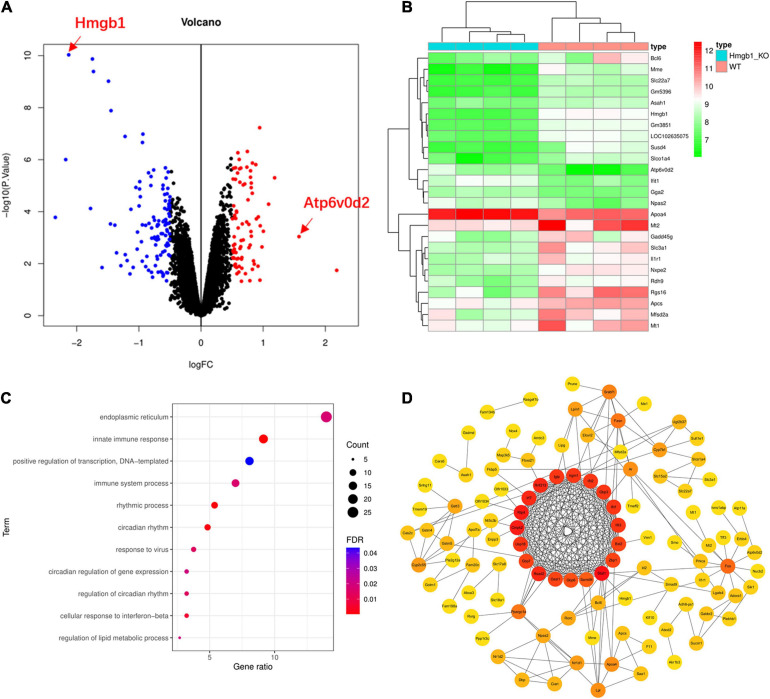
Genes and pathways showed significant changes in Hmgb1^–/–^ mice compared to controls. **(A)** Our *in silico* re-analysis of microarray data from Hmgb1^–/–^ and controls showed 82 upregulated genes and 112 downregulated genes including Atp6v0d2. **(B)** A heatmap showing the comparison of the differentially expressed genes (DEGs) among the Hmgb1^–/–^ and control mice. **(C)** Multiple immune pathways in response to viral infection are enriched in DEGs. **(D)** The protein–protein interaction (PPI) network formed by DEGs.

To further understand the function and role of *HMGB1* in the HBV/HCV infection, we analyzed a microarray dataset from the liver of Hmgb1 knockout mice. Our results yielded 194 differentially expressed genes, including 82 upregulated genes and 112 downregulated genes(log2| FC| > 0.5, *P*-value < 0.05) (Figure 4A and Supplementary Table 6). Consistent with the original report, we did not observe significant changes in expression levels of genes involved in autophagosome formation. However, the key gene Atp6v0d2 functioning at the step of fusion between autophagosome and lysosome showed increased expression in *Hmgb1* KO mice (Figure 4B) (FC = 1.58, *P*-value = 9.11E-04). We further conducted pathway enrichment analysis to examine the signaling pathways affected by Hmgb1 deletion. Five pathways related to virus infection and immune response were significantly enriched among the 194 differentially expressed genes (DEGs), including “innate immune response” and “response to virus” pathways (Figure 4C and Supplementary Table 7). We also constructed a PPI network analysis among the DEGs, revealing 19 significantly associated genes (Figure 4D). Pathway analysis of the significantly associated genes demonstrated significant enrichment of “immune system process”, “defense response to virus”, “negative regulation of viral genome replication” and other immune function-related gene sets (Supplementary Table 8). These findings further suggest that Hmgb1 may regulate hepatitis virus infection.

**FIGURE 5:**
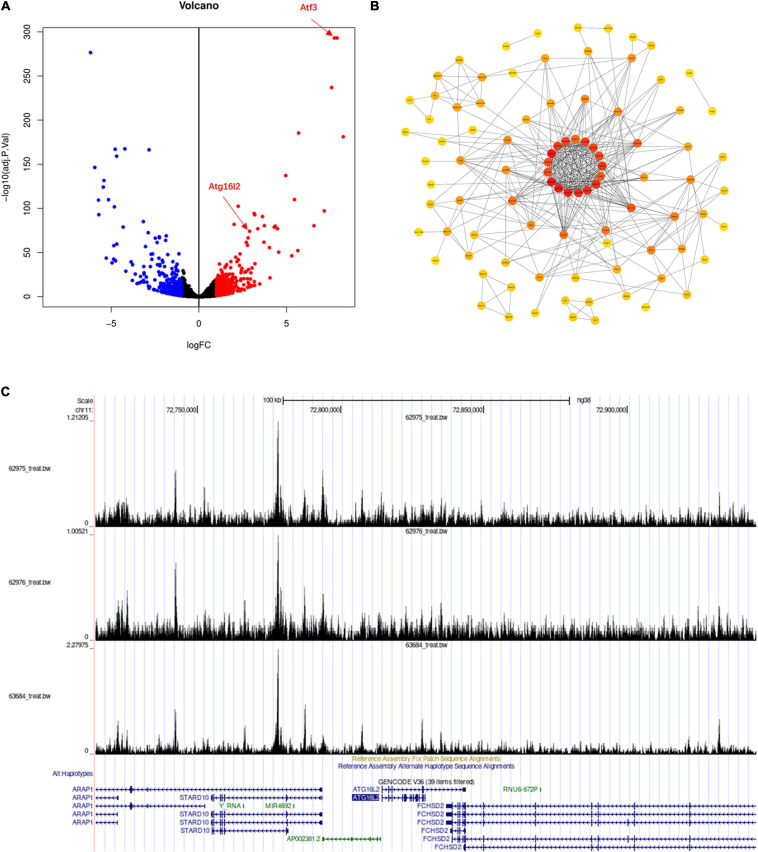
Genes showed significant changes in mouse liver tissue overexpressing *ATF3* compared to controls. We did a differential expression analysis of RNA-seq data from mouse liver tissue overexpressing *ATF3* and controls. **(A)** The results showed 389 significantly upregulated genes and 315 downregulated genes including *Atg16l2*. **(B)** PPI network of genes functioning in immune, endoplasmic reticulum, and mitochondria-related signaling pathways. **(C)** The peaks in the genomic region of human gene *ATG16L2* from ATF3 ChIP-seq data in the ENCyclopedia of DNA Elements.

We also looked into the function of a potential target gene *ATF3* at locus 1q32.3, which exhibited chromatin interaction with GWS SNPs at this locus and showed nominally significant association with these SNPs (Supplementary Tables 4, 5). ATF3 is a negative regulator of autophagy and cellular antiviral signaling by interacting with the promoter of *Atg5*, *Stat1*, *Irf9*, and *Isg15* (Sood et al., 2017). A study also showed that ATF3 promotes hepatitis B virus x mRNA degradation by stimulating Ski2 expression (Shiromoto et al., 2018). We examined a GEO dataset from C57BL/6J mice injected with adeno-associated virus (AAV) expressing human *ATF3* (Xu et al., 2021). We observed the significant upregulation of 389 genes and downregulation of 315 genes in mouse liver tissue (Figure 5A and Supplementary Table 9). Interestingly, the autophagy gene *Atg16l2* showed significant upregulation (logFC = 2.89, adj. *P*-value = 8.07E-75). *Atg16l2* is an isoform of *Atg16l* and can interact with Atg12-conjugated Atg5 to form a complex *in vivo*. Atg16l2 can function as an E3-like enzyme to catalyze the conjugation of LC3 to phosphatidylethanolamine (Ishibashi et al., 2011). The DEGs were enriched in metabolic signaling pathways including mitophagy-related pathways, such as oxidoreductase activity, mitochondrion morphogenesis, and endoplasmic reticulum, and they interconnect with each other (Supplementary Table 10 and Figure 5B). Consistently, we also found enrichment of the organelle membrane pathway, which is involved in diverse biological functions including autophagy (Lin et al., 2021). It is known that viruses may trigger and modulate mitophagy to affect the innate immune response of the host. ATF3 ChIP-seq data from the ENCyclopedia of DNA Elements (ENCODE) showed a strong signal at the genomic region including gene *ATG16L2*, suggesting the likely regulation of *ATG16L2* by ATF3 (Figure 5C).

### Replication of Identified Loci in an Independent Cohort

To validate the GWS loci identified in our study, we conducted a replication study with data extracted from the biobank of CAG, CHOP. The replication cohort of European ancestry after QC filtering includes 67 cases with the diagnosis of HBV or HCV and 649 controls (Supplementary Tables 2, 11). Both the lead SNP rs142568263 at locus 1q32.3 and the top SNP rs76561592 at locus 5p13.3 are associated with HBV/HCV infection in the replication cohort (rs142568263 *P*-value = 0.00874, rs76561592 *P*-value = 0.0139) (Supplementary Table 12); thus, the two loci were replicated with a *P*-value < 0.025. The number of genotyped HBV/HCV cases of East Asian ancestry that we could find is too small for a replication study; however, the peak SNP rs34780238 at locus 13q12.3 is associated with phenotype HBc antigen for hepatitis B virus (phenotype code 23016, *P*-value = 7.757E-5) in UKBB reported in Global Biobank Engine (Stanford, CA)^5^ (McInnes et al., 2019). Variants mapped to *HMGB1* are associated with multiple blood traits, like mean corpuscular volume and eosinophil counts at GWS level, as reported in literature (Astle et al., 2016; Chen et al., 2020). The results from the replication cohort, the supporting evidence from literature, and the known biological function of the candidate genes at each locus suggest that these loci are likely to have *bona fide* HBV/HCV associations.

## Discussion

The HBV–HCV co-infection confers faster disease progression and higher risk of hepatocellular carcinoma development (Mavilia and Wu, 2018). To help to identify patients with high susceptibility to HBV–HCV co-infection, we first conducted a meta-analysis among the Asian population and the European population, respectively, then investigated the potential target genes of each novel GWS locus by functional annotation, and finally we focused on potential target genes *HMGB1* and *ATF3*. We found three loci associated with both HBV and HCV infection. Our results demonstrate perturbation of the Atp6v0d2 gene and the virus immune response pathway upon Hmgb1 deletion. Also, in mouse liver tissue overexpressing ATF3, the influence on Atg16l2 expression and genes in metabolic signaling pathways was demonstrated. Our results suggest the role of target genes at novel GWS loci in connecting autophagy signaling to host immune response from virus invasion.

The *HMGB1* gene encodes a protein high-mobility group box 1, which is highly expressed in different organs, tissues, and cell types including the liver and immune cells. HMGB1 is a chromatin-binding protein that can regulate gene transcription. HMGB1 can also function as a class of damage-associated molecular pattern (DAMP) molecules in response to danger signals such as virus invasion. HMGB1 initiates an inflammatory reaction by interacting with RAGE and TLR4 on neighboring cells and activates the signaling pathway p38/p42/44 MAPK; pJNK; and cJun or p38 MAPK, pERK, and NFκB in different liver cell types (Yang et al., 2020). HMGB1 is involved in the pathogenesis of acute liver injury and chronic liver disease (Gaskell et al., 2018). It was reported that HMGB1 gene 1176G/G genotype is associated with an increased risk of progressive status in HBV infection (Deng et al., 2013). Experimental studies indicated that HCV infection led to the translocation of HMGB1 from the nucleus to the cytoplasm. HMGB1 protein interacts with the HCV genome and increases the replication but not translation of HCV (Yu et al., 2015).

In addition to its function as a DAMP, HMGB1 plays a key role in the regulation of autophagy. HMGB1 can compete with Bcl-2 for binding with Beclin 1 by directly interacting with Beclin 1 and inhibiting phosphorylation of Bcl-2 (Sun and Tang, 2014). Furthermore, oxidation and then cytosolic translocation of HMGB1 increase autophagic flux (Tang et al., 2010). It has been shown that HMGB1 is necessary for hepatic stellate cell activation and participates in HBV-related liver fibrosis progression through its interaction with RAGE and autophagy-inducing effects (Li et al., 2018). HMGB1 is also involved in the pathogenesis of hepatic carcinoma (Chen et al., 2018; Khambu et al., 2018, 2020).

Discrepancy exists regarding the role of HMGB1 in autophagy (Huebener et al., 2014). Here we conducted a re-analysis of microarray data from Hmgb1 KO mice, finding upregulation of Atp6v0d2 and significant expression change of immune response to virus signaling pathway genes. Atp6v0d2 gene encodes ATPase H + transporting V0 subunit D2, which is a subunit of the V0 complex of vacuolar ATPase. Atp6v0d2 is located at lysosomes and endosomes, in addition to vacuoles. Atp6v0d2 interacts with STX17 and VAMP8, which stimulate autophagosome–lysosome fusion, the key final autophagy process (Xia et al., 2019).

Thus, the above evidence suggests that HMGB1 senses HBV/HCV virus infection and transduces that signal to the autophagy pathway and inflammation. The detailed interaction between subsequent pathways downstream of HMGB1 remains to be investigated. Polymorphisms in the genomic locus targeting HMGB1 gene could confer susceptibility to HBV/HCV infection.

With overexpression of ATF3, the potential target gene at locus 1q32.3, an autophagy gene Atg16l2, displayed significant differential expression. Though not essential for canonical autophagy, Atg16l2 is involved in the autophagy process in various cell types. It was shown that knocking down Atg16l2 in human neuroblastoma H4 cells resulted in increased autophagy flux, suggesting the negative regulation of autophagy by Atg16l2 (Lipinski et al., 2010). Another study showed that in the pancreas, where Atg16l2 is highly expressed, knocking down of Atg16l2 led to the accumulation of LC3 and p62, decreased autophagic proteolysis, and slower LC3-II turnover, suggesting the role of ATG16L2 as an adaptor protein in autophagy completion (Li et al., 2013). In addition, with the overexpression of ATF3, genes in a number of metabolic pathways were affected. It is known that metabolism is closely related to both autophagy and immunity (Li et al., 2021). The metabolic status of a cell can have a profound influence on the nature and extent of autophagic induction (Rabinowitz and White, 2010).

In addition to HMGB1 and ATF3, other target genes at the HBV/HCV shared GWS loci are involved in both autophagy and immune signaling pathway to virus infection. Medications targeting these genes are worthy of being investigated for their potential therapeutic role in HBV and HCV infection or co-infection.

## Data Availability Statement

The datasets presented in this study can be found in online repositories. The names of the repository/repositories and accession number(s) can be found in the article/Supplementary Material.

## Ethics Statement

The studies involving human participants were reviewed and approved by the Institutional Review Board at the Children’s Hospital of Philadelphia. Written informed consent to participate in this study was provided by the participants’ legal guardian/next of kin.

## Author Contributions

JL, QX, and HH were responsible for the conception and design of study. XH, JH, HW, and DZ conducted the data analysis and summarization. JG and MM conducted the replication study, data analysis, and interpretation. XH and JG drafted the manuscript. JG, QX, JL, and HH revised the manuscript. All authors have read and approved the manuscript.

## Conflict of Interest

The authors declare that the research was conducted in the absence of any commercial or financial relationships that could be construed as a potential conflict of interest.

## Publisher’s Note

All claims expressed in this article are solely those of the authors and do not necessarily represent those of their affiliated organizations, or those of the publisher, the editors and the reviewers. Any product that may be evaluated in this article, or claim that may be made by its manufacturer, is not guaranteed or endorsed by the publisher.

## Footnotes

1
http://jenger.riken.jp/en/

2
http://string-db.org

3
https://imputation.biodatacatalyst.nhlbi.nih.gov/#!pages/home

4
https://genome.sph.umich.edu/wiki/Minimac4

5
http://gbe.stanford.edu
