# Supplementary material for: Discovery of Novel Host Molecular Factors Underlying HBV/HCV Infection
Source: Front Cell Dev Biol. 2021 Aug 12;9:690882. doi: 10.3389/fcell.2021.690882 (PMC8397444; doi:10.3389/fcell.2021.690882)
Supplement: Supplementary file 1 [file Data_Sheet_1.PDF]

Discovery of Novel Host Molecular Factors Underlying HBV/HCV Infection

Supplementary Figure 1. The genome-wide significant SNPs at the novel loci of 1q32.3 and 5p13.3 and their corresponding target genes are located in the same topologically associating domains (TAD).

A

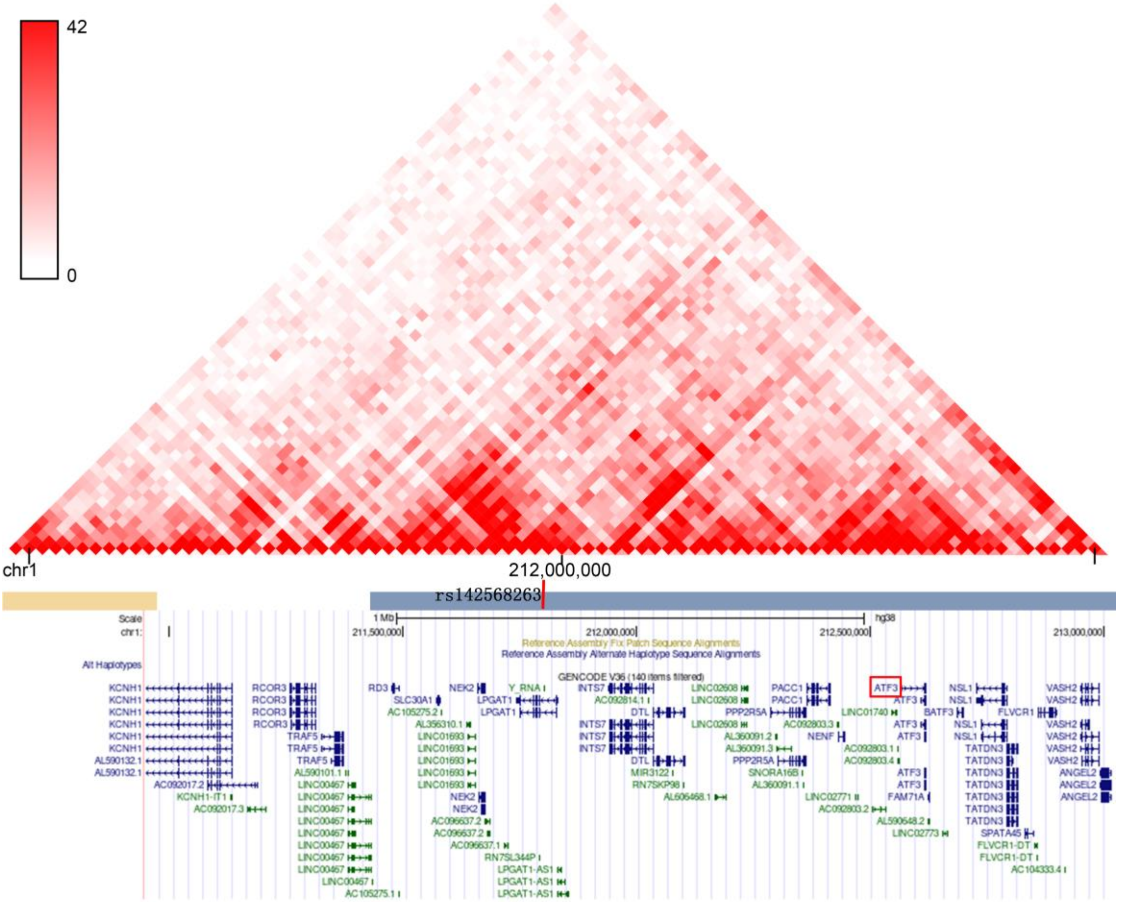

42
0
chr1
212,000,000
rs142568263

B

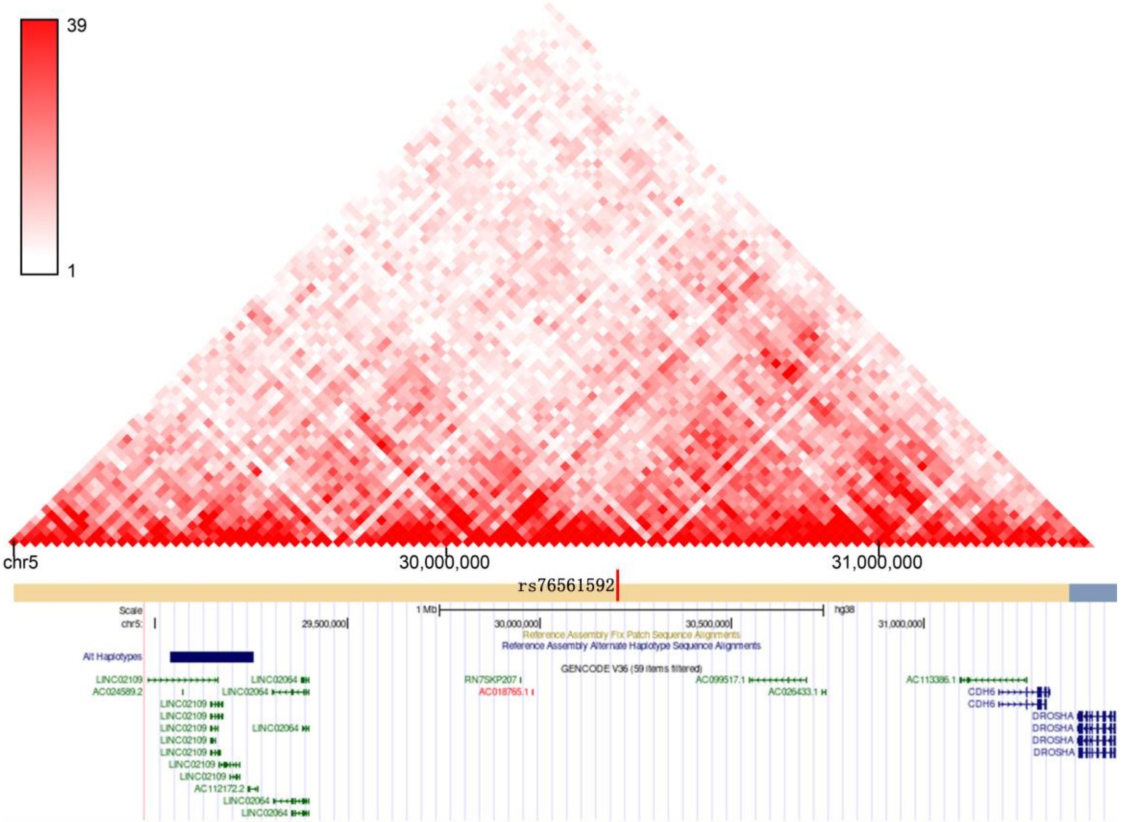

39
1
chr5
30,000,000
31,000,000
rs76561592

Supplementary Table 1. The datasets included in our analysis.

Population	Trait	Description	Case	Control	SNPs before QC	SNPs after QC	SNPs in Meta
East Asian	Chronic hepatitis B	Association result of GWAS for Chronic hepatitis B in the Biobank Japan	1394	211059	8678732	7466610	7463748
East Asian	Chronic hepatitis C	Association result of GWAS for Chronic hepatitis C in the Biobank Japan	5794	206659	8678732	7466650	7463748
European	Viral hepatitis B	Viral hepatitis B	165	413496	28987535	9625308	9610147
European	Viral hepatitis C	Viral hepatitis C	308	413496	28987535	9716108	9610147

Supplementary Table 2. The ICD9 code and the diagnosis name of the cases in the replication cohort.

ICD9	Description
070.3	Hepatitis B Viral hepatitis B without mention of hepatic coma
070.32	Chronic viral hepatitis B without mention of hepatic coma without mention of hepatitis delta
070.41	Acute hepatitis C with hepatic coma
070.44	Chronic hepatitis C with hepatic coma
070.51	Acute hepatitis C without mention of hepatic coma
070.54	Chronic hepatitis C without mention of hepatic coma
070.7	Unspecified viral hepatitis C
070.71	Unspecified viral hepatitis C with hepatic coma

Supplementary Table 3. The potential causal variants overlap with histone marks at gene enhancer or promoter regions.

Population	rsid	chromosome	position	HistoneModification			
East Asian	rs34780238	13	31416073	H3K4me1_Enh			
East Asian	rs3922904	13	31405422	H3K4me1_Enh		H3K27ac_Enh	H3K9ac_Pro
East Asian	rs9579658	13	31405059	H3K4me1_Enh		H3K27ac_Enh	H3K9ac_Pro
European	rs75651594	1	212163342			H3K27ac_Enh	
European	rs142568263	1	211998341	H3K4me1_Enh	H3K4me3_Pro	H3K27ac_Enh	H3K9ac_Pro
European	rs148691694	1	211921465			H3K27ac_Enh	
European	rs150857619	1	212141784				H3K9ac_Pro
European	rs146104002	1	212341169	H3K4me1_Enh		H3K27ac_Enh	H3K9ac_Pro
European	rs76561592	5	30237010				
European	rs77470613	5	30340210				
European	rs77160283	5	29978363		H3K4me3_Pro	H3K27ac_Enh	H3K9ac_Pro

Supplementary Table 4. Genes with nominally significant association with potential causal variants at each novel locus. The eQTL gene of potential causal variant rs150857619 at novel locus 1q32.3 surpassed multiple-testing threshold and is highlighted in bold.(presented in separate excel file)

Supplementary Table 5. Chromatin interactions of credible sets at novel loci. Hi-C genes which also have nominally significant association with GWS SNPs at the locus are highlighted in bold. (presented in separate excel file)

Supplementary Table 6. Genes showed significant differential expression in Hmgb1-/- mice compared to WT mice. (presented in separate excel file)

Supplementary Table 7. Significant results for 194 DEGs in gene-set enrichment analyses. (presented in separate excel file)

Supplementary Table 8. Significant results for 19 significantly associated DEGs in gene-set enrichment analyses. (presented in separate excel file)

Supplementary Table 9. Genes showed significant differential expression in the liver tissue of mice injected with AAV expressing human ATF3 compared to mice without injection. (presented in separate excel file)

Supplementary Table 10. The significant pathway enrichment analysis results of DEGs in mice liver tissue overexpressing ATF3. (presented in separate excel file)

Supplementary Table 11. The characteristics of subjects in the replication cohort after quality control filtering.

	Number	Age range	Median age	Females	Males
Case	67	3-21	12	31	36
Control	649	3-21	12	304	345

Supplementary Table 12. The association statistics of the tested SNPs in the replication cohort of HBV/HCV.

Population	SNP	Chr	Position	Alt/ Ref	Allele freq	N_MISS	HWE_P	Beta	SE	p.value
European	rs142568263	1	211998341	T/C	2.37E-02	0	1	1.17E+00	4.46E-01	8.74E-03
European	rs76561592	5	30237010	G/A	1.61-02	0	1	1.18E+00	4.81E-01	1.39E-02

SNP =rsID of each SNP; Chr=chromosome; Pos=position (hg19); Alt=alternative allele; Ref=reference allele; Allele freq=alternative allele frequency; N_MISS= Number of individuals in the replication cohort missing this SNP; HWE_P= P-value in the test for deviations from Hardy-Weinberg equilibrium
